# The genetics of pre-eclampsia and other hypertensive disorders of pregnancy

**DOI:** 10.1016/j.bpobgyn.2011.02.007

**Published:** 2011-08

**Authors:** Paula J. Williams, Fiona Broughton Pipkin

**Affiliations:** Human Genetics Research Group, School of Molecular and Medical Sciences, University of Nottingham, A Floor West Block, Queen’s Medical Centre, Nottingham NG7 2UH, UK

**Keywords:** pre-eclampsia, genome-wide association study, pregnancy-induced hypertension

## Abstract

Hypertension is the most frequent medical complication occurring during pregnancy. In this chapter, we aim to address the genetic contribution to these disorders, with specific focus on pre-eclampsia. The pathogenic mechanisms underlying pre-eclampsia remain to be elucidated; however, immune maladaptation, inadequate placental development and trophoblast invasion, placental ischaemia, oxidative stress and thrombosis are all thought to represent key factors in the development of disease. Furthermore, all of these components have genetic factors that may be involved in the pathogenic changes occurring. The familial nature of pre-eclampsia has been known for many years and, as such, extensive genetic research has been carried out in this area using strategies that include candidate gene studies and linkage analysis. Interactions between fetal and maternal genotypes, the effect of environmental factors, and epistasis will also be considered.

## Definition of hypertensive disorders of pregnancy

A wide diversity of terminology and diagnostic criteria have been used over the years to classify the hypertensive disorders of pregnancy and define pre-eclampsia. Several internationally recognised definitions are available,^1^ but at present there is no universal classification system or definition of pre-eclampsia. The hypertensive disorders of pregnancy have four defined categories, characterised in Table 1. These are gestational hypertension; pre-eclampsia and eclampsia; superimposed pre-eclampsia and chronic hypertension. This topic is considered further in this issue of *Best Practice and Research Clinical Obstetrics and Gynaecology*.

## Genetic aspects of pre-eclampsia

Clustering of cases of pre-eclampsia within families has been recognised since the 19th century, suggesting a genetic component to the disorder.^2^ Deciphering the genetic involvement in pre-eclampsia is challenging, not least because the phenotype is expressed only in parous women. Furthermore, in complex disorders of pregnancy, it is necessary to consider two genotypes, that of the mother and that of the fetus, which includes genes inherited from both mother and father. Maternal and fetal genes may have independent or interactive effects on the risk of pre-eclampsia. Finally, the heterogeneous nature of the disorder, with a sliding scale of severity, has resulted in differences in the definition of pre-eclampsia used within studies (see above), often with overlap of non-proteinuric gestational hypertension.

Twin studies investigating the relative contribution of genetic versus environmental factors to pre-eclampsia risk, initially yielded disappointing results. They showed that discordance for pre-eclampsia between monozygotic twin sisters was common, suggesting that heritability caused by maternal genes was low.^3^ These early studies were small. More recent investigations, however, using the large Swedish Twin, Medical Birth and Multigeneration Registries have estimated the heritability of pre-eclampsia to be about 55%, with contributions from both maternal and fetal genes. A further study in monozygotic twins^4^ found concordance of pre-eclampsia to be as common as discordance. Evidence from the largest published twin study, which correlated the Swedish Twin Register with the Swedish Medical Register, revealed pre-eclampsia penetrance to be less than 50%, suggesting diversity within models of inheritance.^5–7^

### Pre-eclampsia: a complex genetic disorder

For a small number of families, pre-eclampsia seems to follow Mendelian patterns of disease inheritance,^8^ consistent with a rare deleterious monogenic variant or mutation with high penetrance. For most of the population, however, pre-eclampsia seems to represent a complex genetic disorder, and occurs as the result of numerous common variants at different loci which, individually, have small effects but collectively contribute to an individual’s susceptibility to disease. Environmental exposures, including age and weight, also determine whether these low penetrant variants result in phenotypic manifestation of the disease. It is likely that no single cause or genetic variant will account for all cases of pre-eclampsia, although it is possible that different variants are associated with various subsets of disease (e.g. pre-eclampsia combined with intrauterine growth restriction). Complex genetic disorders affect a high proportion of the population, representing a large burden to public health. New approaches to susceptibility gene discovery have emerged to address this challenge. Unfortunately, early diagnosis would only permit closer focus on routine antenatal care, as at present no intervention other than delivery has been shown to alter the course of pre-eclampsia.

### Determining susceptibility to pre-eclampsia

The need to assess both the maternal and the fetal genotype is clear. The role of the placenta in the primary pathogenesis of the disorder indisputably indicates a fetal contribution to susceptibility to the disorder.^9^ Reports of severe, very early-onset pre-eclampsia in cases of fetal chromosomal abnormalities such as diandric hydatifidiform moles of entirely paternal genetic origin^10^ are consistent with a role for paternally inherited fetal genes in the determination of clinical phenotype. This is supported by epidemiological studies reporting a higher rate of pre-eclampsia in pregnancies fathered by men who were themselves born of pre-eclamptic pregnancies.^11^ The occurrence of pre-eclampsia in daughters-in-law of index women^9^ further supports a genetic contribution from both parents. The genetic conflict hypothesis states that fetal (paternal) genes will be selected to increase the transfer of nutrients to the fetus, whereas maternal genes will be selected to limit transfer in excess of a specific maternal optimum.^12^ Fetal genes are predicted to raise maternal blood pressure in order to enhance the uteroplacental blood flow, whereas maternal genes act the opposite way. Endothelial dysfunction in mothers with pre-eclampsia could, therefore, be interpreted as a fetal attempt to compensate for an inadequate uteroplacental nutrient supply.

As the phenotype is apparently only expressed during pregnancy, identification of ‘susceptible’ men is impossible. Most genetic studies of pre-eclampsia have focused on maternal genotypes only. The Genetics of Pre-eclampsia consortium highlighted the need to include analysis of all contributing genotypes, and carried out transmission disequilibrium testing in maternal and fetal triads.^13^ Understanding the contribution of the fetal genotype will require large sample sizes, with the development of algorithms to determine the relative contribution from mother and fetus. Furthermore, the decreased incidence of pre-eclampsia in second and subsequent pregnancies hampers analysis of the contribution of the fetal genotype.

### Candidate gene approach

The candidate gene approach has been widely used in pre-eclampsia, and largely focuses on the maternal genotype. In this method, a single gene is chosen as the candidate for investigation based on prior biological knowledge of the pathophysiology of pre-eclampsia. The choice is strengthened if the gene lies within a region identified by linkage studies. A case-control design is usually used, comparing the frequencies of allelic variants in women with pre-eclampsia and normotensive pregnancies. Such studies need careful definition of inclusion criteria for cases and controls, and subtle ethnic stratification of groups must be avoided. Such performance characteristics of the genotyping assays as the rate of mis-genotyping, and the quality assurance methods used, should be clearly stated, but this is rarely done. Over 70 biological candidate genes have been examined, representing pathways involved in various pathophysiological processes, including vasoactive proteins, thrombophilia and hypofibrinolysis, oxidative stress and lipid metabolism, endothelial injury and immunogenetics.^14^ In common with the experience in other genetically complex disorders, results from candidate gene studies have been inconsistent, and no universally accepted susceptibility gene has been identified. Although this may, in part, be attributed to variation within populations, a more important factor is the small size of most of the candidate studies, which have been underpowered to detect variants with small effects. As there are more than 20,000 genes and 10 million single nucleotide polymorphisms (SNP) available, multiple testing will inevitably result in numerous results that achieve P values of less than 0.05. The development of robust statistical techniques for the minimisation of both false positive and false negative results is an important area.^15,16^ Only in recent years, as susceptibility genes for other complex disorders have been reported, has the small effect size of individual genetic variants become apparent, the majority increasing the risk of disease by less than 50%. A further limitation of the candidate gene approach is its reliance on the generation of an *a-priori* hypothesis based on our current incomplete knowledge of the pathophysiology of the disorder. The candidate genes studied belong to different groups according to their functional properties and plausible role in the pathophysiology (Table 2).

#### Thrombophilia

A successful pregnancy requires the development of adequate placental circulation. It is hypothesised that thrombophilias may increase the risk of placental insufficiency because of placental micro-vascular thrombosis, macro-vascular thrombosis, or both, as well as effects on trophoblast growth and differentiation.^17^ Abnormalities of the clotting cascade are well documented in women with pre-eclampsia.^18^ The endothelial damage of pre-eclampsia is associated with an altered phenotype from anticoagulant to procoagulant and decreased endothelially mediated vasorelaxation. It is possible that this phenotype is present before pre-eclampsia in pregnancy, or it may develop as a consequence of damage initiated during placentation. Furthermore, a subset of women develop frank thrombocytopaenia, often in association with haemolysis, elevated liver enzymes and low platelet count (HELLP) syndrome. Association of the three most widely studied thrombophilic factors, factor V Leiden (*F5*), methylenetetrahydrofolate (*MTHFR*) and prothrombin (*F2*), with pre-eclampsia has been shown; however, several studies have also shown contradictory results.^14^ A recent meta-analysis indicated a two-fold increase in risk for pre-eclampsia associated with 1691G>A mutation in *F5*, but no associations were found for *MTHFR* or *F2.*^19^ To date, the number of studies showing no association with pre-eclampsia for these three genes is much higher than the number confirming association. Association with the inhibitor of fibrinolysis plasminogen activator factor-1 gene has also been reported; however, replication attempts have failed.^20–22^

#### Haemodynamics and endothelial function

The renin-angiotensin system (RAS) is important for regulating the cardiovascular and renal changes that occur in pregnancy. Several studies have implicated the RAS in the pathophysiology of pre-eclampsia.^23^ As such, genes in the RAS have been considered as plausible candidates for pre-eclampsia. Angiotensin-converting enzyme (*ACE*), angiotensin II type 1 and type 2 receptor (*AGTR1, AGTR2*), and angiotensinogen (*AGT*) have all been studied extensively in pre-eclampsia. Recent meta-analyses have identified the T allele of *AGT* M235T as increasing the risk of developing pre-eclampsia by 1.62 times and similar increases in disease risk have been found in *AGT* and the angiotensin-converting enzyme I/D polymorphism.^24^ A rare functional polymorphism in *AGT*, which results in replacement of leucine by phenylalanine at the site of renin cleavage, has been reported in association with severe pre-eclampsia.^25^

Endothelial nitric oxide synthase 3 (*eNOS3*), which is involved in vascular remodelling and vasodilation, has been shown to have reduced activity in pre-eclampsia^26^ Association studies in different ethnic populations, however, have yielded both positive and negative findings. A meta-analysis investigating the E298D polymorphism, which had initially been associated with pre-eclampsia in Colombian women, failed to find increased risk.^24^ Vascular endothelial growth factor (*VEGF*) is important for endothelial cell proliferation, migration, survival and regulation of vascular permeability. The number of studies that have investigated SNP in the genes involved in the *VEGF* system is small. Two polymorphisms in *VEGF*, 405G>C and 936C>T, were found to be associated with the severe form of pre-eclampsia in two small studies, but cannot at present be considered as major risk factors.^27,28^

#### Oxidative stress and lipid metabolism

Oxidative stress plays a central role in the pathogenesis of pre-eclampsia. Maternal perfusion of the placenta does not occur until towards the end of the first trimester,^29^ when a rapid increase in local oxygen tension takes place, and the probable occurrence of a period of hypoxia–reperfusion until stability is reached. This is accompanied by increased expression and activity of such antioxidants as glutathione peroxidase, catalase and the various forms of superoxide dismutase.^30^ If this antioxidant response were reduced, then the cascade of events leading to impaired placentation could be initiated. Evidence for reduced antioxidant activity in pre-eclampsia has recently been reviewed.^31^ Genes involved in the generation or inactivation of reactive oxygen species, if defective, could increase endothelial dysfunction via lipid peroxidation, which has been a candidate causative agent for the endothelial damage of pre-eclampsia for more than 20 years.^32^ Despite the strong correlation between oxidative stress and pre-eclampsia, only a small handful of genes have been investigated. Functional polymorphisms in the gene for microsomal epoxide hydrolase (*EPHX*) that catalyses the hydrolysis of certain oxides and may produce toxic intermediates that could be involved in pre-eclampsia, and glutathione S-transferase (*GST*), an antioxidant capable of inactivating reactive oxygen species, have shown associations. Conflicting results, however, have also been reported.^33–36^

Abnormal lipid profiles associated with the lipid peroxidation caused by oxidative stress are also characteristic of pre-eclampsia. Lipoprotein lipase (*LPL*) and apolipoprotein E (*ApoE*) are the two major regulators of lipid metabolism, abundantly expressed in placenta, and have therefore been proposed as possible candidate genes.^37,38^ A recent study using bioinformatic analysis identified altered glycosylation of circulating ApoE isoforms in pre-eclampsia.^39^ A deglycosylated basic ApoE isoform was increased in pre-eclampsia, and an acidic ApoE sialyated isoform was decreased. Functionally, this might increase the risk of developing placental atherotic changes. The most promising genetic variant in this context is a mis-sense mutation, Asn291Ser, in *LPL* which correlates with lowered *LPL* activity and increased dyslipidaemia in two separate studies. Again, others have failed to replicate these findings.^38,40,41^ The fetal genotype of these two genes has also been reported to contribute to the metabolism of the maternal lipoproteins.^37^

#### Immune system

The maternal immune response to pregnancy is crucial in determining pregnancy outcome and success. The increased incidence of pre-eclampsia in primiparous women, especially those at either end of the childbearing age range, indicates a strong association between immune factors and pre-eclampsia.^42^ However, the protective effect of multiparity is lost with change of partner. Advances in assisted reproductive technology are also posing new challenges to the maternal immune system. The use of donated sperm or eggs increases the risk of pre-eclampsia three-fold.^43^

#### Human leucocyte antigen

Trophoblast cells express an unusual repertoire of histocompatibility antigens, comprising human leucocyte C, E and G class antigens (HLA-C, HLA-E, HLA-E), of which only HLA-C displays marked polymorphism. The expression of HLA on the invading cytotrophoblast is important, as these interact with killer immunoglobulin, such as receptors (KIR) expressed on maternal uNKs and cytotoxic T-lymphocytes, down-regulating their cytolytic activity and stimulating the production of cytokines needed for successful placentation. Multiple highly homologous KIR genes map to chromosome 19q, probably arising from ancestral gene duplications, and the two main resulting gene clusters have been classified as haplotypes A and B. The A group codes mainly for KIR, which inhibit natural killer cells, whereas the B group has additional stimulatory genes.^44^ Pre-eclampsia is more frequent in women who are homozygous for the inhibitory A haplotypes (AA) than in women homozygous for the stimulatory B haplotypes (BB). The effect is strongest if the fetus is homozygous for the HLA-C2 haplotype.^45^ Alteration in KIR interaction on uNK cells with HLA-C on interstitial trophoblast alters the decidual immune response, resulting in impaired extravillous trophoblast invasion and deficient spiral artery remodelling, associated with pre-eclampsia.

An association of HLA-G, which displays limited polymorphism, with pre-eclampsia, has also been reported. A possible association between the presence of the HLA-G allele G*0106 in the placenta and an increased risk of pre-eclampsia has been identified in two small studies.^46,47^ these were underpowered, however, and further studies using larger cohorts of mothers and babies are needed to replicate these results. HLA-G variants foreign to the mother may lead to histo-incompatibility between mother and child. A maternal rejection response to the semi-allogeneic fetus may represent one of the pathways involved in the development of pre-eclampsia.

A number of pro-inflammatory cytokines have also been investigated for possible associations with pre-eclampsia. Excessive release of tumour necrosis factor alpha (TNFα) has been implicated owing to its contribution to endothelial activation, which in turn could contribute to maternal symptoms.^48^ Interestingly, in pregnant rats, TNF induces hypertension, a response not seen in non-pregnant rats.^49^ Furthermore, plasma levels of TNFα are significantly higher in women with pre-eclampsia than matched controls.^50^ TNFα is also involved in the production of reactive oxygen species and subsequently oxidant mediated endothelial damage. The most frequently studied variant in pre-eclampsia is the –308G>A transition in the promoter region, which is associated with increased levels of TNFα production and an increased risk for pre-eclampsia linked disorders, including type 2 diabetes, coronary artery disease and dyslipidaemia.^51,52^ However, a meta-analysis from 2008 combined 16 studies investigating this promoter SNP, but failed to detect a significant association to pre-eclampsia.^53^

Interleukin-10 (*IL-10*) has also been implicated in the pathogenesis of pre-eclampsia by enhancing the inflammatory response towards trophoblast cells resulting in reduced invasion and remodelling of the spiral arteries.^54^ Expression of *IL-10* is reduced in pre-eclamptic placentae.^55^ Studies investigating associations of variants of the gene and pre-eclampsia, however, have yielded conflicting results.^56–58^ Associations have also been detected for two additional inflammatory genes, interleukin-1α (*IL-1α*) and the interleukin 1 receptor anatagonist (*IL1Ra*) in relatively small studies, but few studies have addressed the role of polymorphisms in these genes so far.^59,60^

#### Antioxidant enzymes

A large family of cytosolic glutathione-s-transferases (GST) exists, and the P class is highly expressed in the human placenta. Several relatively small case-control studies of polymorphisms in this family in relation to pre-eclampsia have failed to identify any significant effect of several GST polymorphisms studied individually. However, a cumulative effect of the number of polymorphisms in various biotransformation enzymes, including GST, which would result in decreased antioxidant capacity, has been reported.^61^ Intriguingly, the use of semi-quantitative polymerase chain reaction on a small data set identified using serial analysis of gene expression profiles, seems to identify a specific molecular signature for HELLP, which includes decreased expression of GST P1.^62^

Remarkably, few studies of possible functional polymorphisms in antioxidant enzyme systems have been reported. The 242C>T polymorphism in exon 4 of the gene for the p22phox subunit of NADPH/NADH oxidase (*CYBA*), which is part of the cascade of superoxide generation, has been reported as showing no evidence of an association with either pre-eclampsia or HELLP and pre-eclampsa.^63^ A small preliminary study of the Ala40Thr polymorphism of the superoxide dismutase 3 gene (*SOD3*), which has been associated with insulin resistance, reported a significant excess of the mutant allele in women with severe intrauterine growth restriction.^64^

## Genome-wide screens

The human genome consists of about 25,000 genes, including a significant proportion of unknown function. With the advent of microarray genotyping technologies, screening of the entire genome is now possible. Genome-wide screening is an ‘agnostic’ approach that is not limited by current biological functional knowledge used in the candidate gene approach, with the prospect of providing novel insights into the disease process.

### Genome-wide linkage screens

Genome-wide linkage screens (GWLS) have been very successful in identifying causal variants with high penetrance in monogenic disorders, but this method has limited power to detect genes with small effect size in complex genetic disorders. In pre-eclampsia the lack of a recognisable phenotype in men or non-parous women, and uncertainty of the mode of inheritance, has made it difficult to carry out conventional linkage analysis. GWLS have been carried out using affected sib-pair analysis, analysing the segregation of genetic markers (microsatellite alleles) between index women and their affected siblings. This method has been extended to more distant relationships using affected pedigree member analysis. Linkage analysis can only identify relatively large regions (typically tens of cM), which can contain hundreds of genes, including many which are biologically plausible.

GWLS of pre-eclampsia has revealed significant linkage on chromosomes 2p13,^65^ 2p25, and 9p13.^66^ Suggestive linkage has been identified at other loci on chromosomes 2q, 9p, 10q, 11q and 22q^67,68^ (Table 3)^69^. Disappointingly, no significant or suggestive locus has been replicated in another GWLS. Possible explanations include population variations and differences in the density of microsatellite marker panels, but limited statistical power is a major factor in failure to replicate GWLS results in complex disorders. A meta-analysis of the results of five GWLS yielded modest evidence for linkage at several loci, but cautioned that insufficient data were available for conclusive results.^70^

### Positional candidate genes

#### Activin A receptor type IIA

Associations between positional candidate genes on the 2q22-23 susceptibility locus identified in GWLS have been examined further by both Norwegian, Australian and New Zealand groups. Activin A receptor type IIA (*ACVR2A*) was identified as a strong positional candidate on this locus. *ACVR2A* is a key receptor for the cell signalling protein activin A, an important regulator of human pregnancy. Circulating levels of activin A are increased in pre-eclamptic pregnancies, suggesting its use as a potential biomarker of pre-eclampsia.^71^ Significant associations with pre-eclampsia were found for four *ACVR2A* SNP in a study of over 1100 pre-eclamptic women and 2200 normotensive controls.^72^ The influence that these variants have on the expression or function of *ACVR2A* is under investigation. However, the *ACVR2A* association with pre-eclampsia was not confirmed in a study of 74 affected families from Australia and New Zealand.^73^ Owing to the strong biological involvement of Activin A in the establishment of pregnancy, this gene still remains a priority for further adequately powered studies.

#### ROCK2

*ROCK2*, the gene encoding rho-associated coiled-coil protein kinase 2, lies within the pre-eclampsia linkage peak on chromosome 2p25 identified in a GWLS of Finnish families.^66^ Interestingly, this gene has been implicated in essential hypertension. *ROCK2* is widely expressed in smooth muscle cells, and a suggested role in vasoconstriction has been confirmed in a number of animal models.^74,75^ In addition, *ROCK2* is expressed by syncytiotrophoblast cells of the placenta, and expression is reportedly up-regulated in pre-eclampsia.^76^ The Finnish group compared 10 polymorphisms in *ROCK2* in 340 unrelated cases with matched normotensive controls, but did not show any association between these variants and pre-eclampsia. The study was powered to detect a genetic effect of 1.6; a larger study is required before *ROCK2* can be ruled out as a susceptibility gene for pre-eclampsia. the results of the earlier GWLS of Finnish families, however, suggest a gene with a substantial effect size, possibly owing to other genes at the 2p25 locus.

#### ERAP1 and ERAP2

Detailed study of susceptibility genes at the 5q15 locus following GWLS in separate Australian and Norweigan women identified *ERAP1* and *ERAP2* as being significantly-associated with pre-eclampsia.^77^ *ERAP1* degrades angiotensin II to angiotensin III, and has been linked with blood-pressure control in non-pregnant women with hypertension.

### Genome wide association screening

Genome-wide association screening (GWAS) makes use of the abundant SNP in the human genome. As their name indicates, SNP are polymorphisms involving a change in a single base. These are commonly biallelic (two possible alleles), and occur within gene coding and, more commonly, non-coding regions. Over 10 million common SNP have been identified within the human genome and, in theory, any one of these might affect gene function or expression, and influence susceptibility to disease. Genotyping of all 10 million SNP would not only be time consuming but also prohibitively expensive using current technologies. The phenomenon of linkage disequilibrium – the lack of independence between the alleles of SNP in close proximity – makes it possible to genotype a smaller number of representative tagSNP and infer the genotype of adjacent SNP. Typically, genotyping between 300,000 and 1 million carefully selected tagSNP will capture most variation in the human genome. It is then possible to focus on a region showing association with disease to determine whether this tagSNP, or more likely another SNP in linkage disequilibrium, is the causal variant. This often requires extensive re-sequencing of the region to identify all polymorphisms.

Although GWAS offers an exciting way forward for identifying susceptibility variants in complex disorders, a number of conditions must be met to make the screen robust and the results of value. The odds ratio of disease conferred by a single SNP is generally low, and this means that only large, well-powered studies will identify such SNP. Two thousand cases is regarded as the minimum for GWAS. Furthermore, owing to the large number of statistical comparisons carried out in GWAS, a stringent threshold for declaring statistical significance is required.^78^ A consensus level is *P* < 5 × 10^−7^. The use of population-based control samples has been validated through GWAS carried out as part of the Wellcome Trust Case Control Consortium. However, such populations are only suitable if the disorder has a relatively low incidence in the general population.

GWAS have identified at least 2000 common variants that seem to be associated with common diseases or related traits, hundreds of which have been convincingly replicated. Successful GWAS have identified association signals in a number of complex diseases, including bipolar disorder, coronary artery disease and type 2 diabetes.^78^ Only modest effect sizes have been observed in many well-replicated GWAS loci (odds ratio less than 1.2), emphasising the need for appropriately large sample sizes and for further functional studies to elucidate the exact biological mechanism involved in disease pathology. GWAS to identify susceptibility DNA variations associated with pre-eclampsia are under way in a number of centres at the time of writing, and the results are awaited with interest.

## The role of genetic imprinting in pre-eclampsia

Several genes in conserved clusters are expressed from only the maternal or the paternal allele, the other allele being genetically silenced (’imprinted’). Imprinted genes are involved in regulating trophoblast growth and fetal development.^79^ Imprinted genes associated with disease show an unusual mode of inheritance, as mutant genes have an effect on the phenotype only if they come from the parent from which they are expressed. This may explain some conditions that seem to be heritable but show an inconsistent pattern in affected families.

Epigenetic modification, including imprinting, has been implicated in the defective trophoblast invasion characteristic of pre-eclampsia. The p57kip2 mouse model of pre-eclampsia is heterozygous for deficiency of the maternally expressed (paternally imprinted) *Cdkn1c* gene.^80^ Although this gene is also imprinted in humans, its role in human disease pathology is unclear as 11p15, the location of the human *CDKN1C* gene, does not display linkage with pre-eclampsia. A GWLS of Dutch families affected by pre-eclampsia showed linkage to chromosome 10q22.1 with inheritance by the affected sisters of shared maternal alleles in all families, suggesting a parent-of-origin effect. Furthermore, these same loci had down-regulated expression of two gene clusters in hydatidiform molar placentae of androgenetic origin.^81^ These included the gene encoding the transcription factor STOX1, which seems to be involved in the normal transition of trophoblast cells from an invasive to a non-invasive phenotype. Support for a functional role for STOX1 in trophoblast maturation has come from studies in a choriocarcinoma cell line over-expressing STOX1. The alterations in the transcriptome of these cells correlated strongly with those observed in pre-eclamptic placentae.^82^ A common single nucleotide polymorphism within the *STOX1* gene alters an amino acid at the DNA binding site, making *STOX1* an attractive candidate for pre-eclampsia. However, subsequent studies have challenged the imprinted status of *STOX1*, and case-control studies have failed to show an association between *STOX1* and pre-eclampsia.^83–85^ Further studies of this intriguing gene are required to clarify its role, if any, in pre-eclampsia.

The *H19* gene, in which the paternal allele is imprinted and the maternal allele is expressed, has also been implicated in pre-eclampsia owing to its role in regulating the growth and development of the embryo and differentiation of cytotrophoblast cells.^86^ In normal placentae, biallelic expression of *H19* was observed in the first trimester, but as pregnancy progressed, the paternal allele was silenced. In contrast, biallelic expression was observed in a significant proportion of pre-eclamptic placentae obtained at delivery. The investigators suggested that *H19* is imprinted dynamically during pregnancy, with loss of imprinting associated with pre-eclampsia. Further work is needed to confirm these initial findings.

## Gene interactions

Epistasis, the modification of expression of one gene by one or several other genes, is believed to be an important genetic contributor to complex diseases, including pre-eclampsia. Although the examination of epistatic interactions is essential to our understanding of the genetic basis of pre-eclampsia, it presents substantial statistical challenges, particularly in the analysis of genome-wide data, owing to the vast number of possible interactions. Robust statistical tools for the study of epistatic interactions are currently under development.

In addition to gene–gene interactions, the clinical phenotype of affected individuals is also influenced by gene–environment interactions.^87^ A lack of complete concordance for pre-eclampsia in identical twins is a clear indication of the effect that environmental factors may play in determining the clinical phenotype^3,88^ Environmental variables associated with pre-eclampsia include diet, smoking, alcohol and obesity. These are able to alter the rate of gene transcription and translation, which may be one mechanism by which they modify disease risk. Collection of robust data on environmental exposures is needed to allow the effect of the environment on the incidence of pre-eclampsia to be fully assessed. Ideally, such studies should make use of prospective cohorts of pregnant women such as the SCOPE and Norwegian Mother and Child Cohort Study biobanks.^89,90^

## The future

Researchers are acknowledging the need for the formation of large DNA sample collections, and collaboration between groups in order to form such collections. We will be closer to identifying DNA variations that are involved in pre-eclampsia when the following takes place: sample banks are formed, and sample sizes are provided with high statistical power that allow us to identify polymorphisms with small effects to carry out further subgroup analysis. Furthermore, studies that can elucidate the relative involvement and interaction of maternal and fetal genotypes, together with information on possible environmental effects, will be beneficial.

Understanding how genes are involved in pre-eclampsia will enable us to identify women at high risk and thus target specialised antenatal care to this group. However, experience with Type 2 diabetes shows genetic testing to be an expensive method of predicting disease risk, with markers such as body mass index and family history found to be of more practical predictive value. Identification of novel pharmaceutical targets and additional therapies may be additionally be aided by knowing the genetic component of pre-eclampsia.

## Conclusion

Epidemiological studies clearly confirm a genetic component to pre-eclampsia. Numerous candidate genes have been studied that fall into groups based on their proposed pathological mechanism, including thrombophilia, endothelial function, vasoactive proteins, oxidative stress and lipid metabolism and immunogenetics. It is expected that no one gene will be identified as the sole risk factor for pre-eclampsia, as in the general population pre-eclampsia represents a complex genetic disorder. Interactions between numerous SNP either alone or with combination with predisposing environmental factors, are most likely underpin the genetic component of this disorder. We must be cautious in our approach to genetics and acknowledge that we are still in the infancy of this research. Following on from GWAS, further fine mapping studies to delineate SNP that are causal from those that are in linkage disequilibrium, followed by functional laboratory studies will be required. Only when we have a better understanding of how the environment interacts with genes will we be in a better position to target treatment for women, for example knowing that women with a certain genotype will benefit from losing weight, enabling us to yield clinical benefit.

**Practice points**

At present no genetic test is available to predict pre-eclampsia. The lack of a predictive test can be overcome by careful monitoring and assessment of women, especially those in high-risk groups, including:
  • Those at either end of the reproductive age spectrum
  • Obesity
  • Black ethnicity
  • Primiparity
  • Previous history of pre-eclampsia
  • Multiple pregnancy
  • Pre-existing medical conditions: renal disease, insulin-dependent diabetes, autoimmune disease, antiphospholipid syndrom

**Research agenda**

• The potential benefits of individual genomewide screening for healthcare and treatment options.
• The importance of fetal development and later health in adulthood (e.g. Developmental Origins of Health and Disease; DoHAD).
• The maternal immune system response to pregnancy.

## Conflict of interest

None declared.

## Figures and Tables

**Table 1:** Commonly used diagnostic criteria and classification of hypertensive disorders of pregnancy. The pregnancy-specific conditions may also be diagnosed where diastolic pressure exceeds 90 mmHg but systolic pressure is < 140 mmHg. It is usual to exclude the diagnosis if hypertension is recorded only during labour.

Classification	Diagnostic criteria
Gestational hypertension	Hypertension: blood pressure ≥ 140/90 mmHg after 20th week of pregnancy in a previously normotensive woman.
Pre-eclampsia	Hypertension: blood pressure of ≥ 140/90 mmHg after 20th week of pregnancy in a woman who was previously normotensive.
	Proteinuria: urinary excretion ≥ 300 mg/L or 500 mg/24 h in the absence of urinary tract infection.
Eclampsia	Occurs in a woman with pre-eclampsia.
	Characterised by seizures not attributed to other causes.
Superimposed pre-eclampsia	Chronic hypertension with development of proteinuria during pregnancy.
Chronic hypertension	Hypertension present before 20th week of pregnancy, persistent for more than 6 weeks postpartum, or both.

**Table 2:** Predominant functional candidate genes studied in pre-eclampsia.

Pathophysiological mechanism group	Gene name	Gene symbol	Predominant polymorphism investigated
Thrombophilia	Factor V Leiden	*F5*	506Gln>Arg
	Methylenetetrahydrofolate	*MTHFR*	C667T
	Prothrombin	*F2*	G20210A
	Plasminogen activator factor-1	*SERPINE1*	I/D promoter
	Integrin glycoprotein IIIa	*GPIIIA*	C98T
Endothelial function	Endothelial nitric oxide synthase 3	*eNOS3*	298Glu>Asp
	Vascular endothelial growth factor receptor 1	*VEGFR1*	TG repeat
	Vascular endothelial growth factor	*VEGF*	C936T
Vasoactive proteins	Angiotensinogen	*AGT*	235Met>Thr
	Angiotensin converting enzyme	*ACE*	I/D intron 16
Oxidative stress and lipid metabolism	Apolipoprotein E	*APOE*	C866T
	Microsomal epoxide hydrolase	*EPHX*	113Tyr>His
	Glutathione S-transferase	*GST*	A313G
Immunogenetics	Tumor necrosis factor α	*TNF*	G-308A
	Interleukin 10	*IL10*	G1082A

I/D, insertion/dilution.

**Table 3:** Genome-wide linkage scans to identify susceptibility loci for pre-eclampsia carried out over the past 10 years.^a^

Country	Study size	Number of microsatellite markers used	Chromosome loci identified	cM	Logarithm of the odds (LOD) score
Iceland	124 families (343 women)	440 microsatellite markers (spacing ∼9cM)	2p13	94.05	4.77
Australia/New Zealand	34 families (366 women)	400	2q23	144.7	2.58
			11q23	121.3	2.02
The Netherlands	38 families (332 women)	292 (spacing ∼11.8cM)	10q22	93.9	2.38
			22q12	32.4	2.41
Finland	15 families (174 women)	435 (spacing ∼10cM)	2p25	21.70	2.51
			9p13	38.90	2.22
			4q32	163.0	2.96
			9p11	49.9	2.20

a A logarithm of the odds (LOD) score > 3.6 (*P* value < 0.00002) indicates genome-wide significance; an LOD score between 2.2 and 3.6 (*P* < 0.0007) indicates suggestive linkage; and an LOD score between 0.6 (*P* < 0.05) and 2.2 (*P* < 0.01) are nominal.^69^
